# Poster Session II - A266 A REAL-WORLD CANADIAN RETROSPECTIVE STUDY OF DRUG PERSISTENCE OF MIRIKIZUMAB IN ADULT PATIENTS WITH MODERATELY TO SEVERELY ACTIVE ULCERATIVE COLITIS

**DOI:** 10.1093/jcag/gwaf042.266

**Published:** 2026-02-13

**Authors:** V Jairath, R Panaccione, T Bessissow, B Bressler, G Ng, J Glass, M Braun, T Movsessian, B Feagan

**Affiliations:** Western University, London, ON, Canada; University of Calgary, Calgary, AB, Canada; McGill University Health Centre, Montreal, QC, Canada; The University of British Columbia, Vancouver, BC, Canada; IQVIA Canada, Kirkland, QC, Canada; Eli Lilly Canada Inc, Toronto, ON, Canada; Eli Lilly Canada Inc, Toronto, ON, Canada; Eli Lilly Canada Inc, Toronto, ON, Canada; Western University, London, ON, Canada

## Abstract

**Background:**

While mirikizumab (MIRI) is an effective therapy for ulcerative colitis (UC), there are limited real-world data describing patient characteristics and treatment persistence.

**Aims:**

We describe patient characteristics and drug persistence in the real-world setting for patients enrolled in the LillyPlus^®^ Patient Support Program (PSP) across Canada. Additionally, we evaluated results by prior advanced therapy (AT) use.

**Methods:**

The study included adult patients (≥18 years old) with moderate-to-severe UC who were enrolled in the PSP and received at least one dose of MIRI (index date) between October 26, 2023, and April 28, 2025. Persistence was defined as remaining on MIRI (induction: 300 mg intravenously every 4 weeks [Q4W] for 12 weeks with the possibility of extended induction; maintenance: 200 mg subcutaneously Q4W thereafter), and was assessed up to 6 months post-index using Kaplan-Meier analysis.

**Results:**

A total of 774 eligible patients were included; the mean (standard deviation) age was 46.5 (17.4) years, 87.0% were AT experienced, and 51.9% were female. Age and gender proportions were similar regardless of prior AT experience. At 6 months, drug persistence rate was 80.9% (432 patients persistent, 220 censored), with rates slightly higher in AT-naïve than in AT-experienced patients (85.1% vs 80.4%; Figure 1).

**Conclusions:**

This real-world study reports relatively high treatment persistence to MIRI at 6 months in both AT-naïve and AT-experienced subgroups. Long-term analysis of PSP data beyond 6 months will further elucidate treatment persistence in this UC patient cohort.

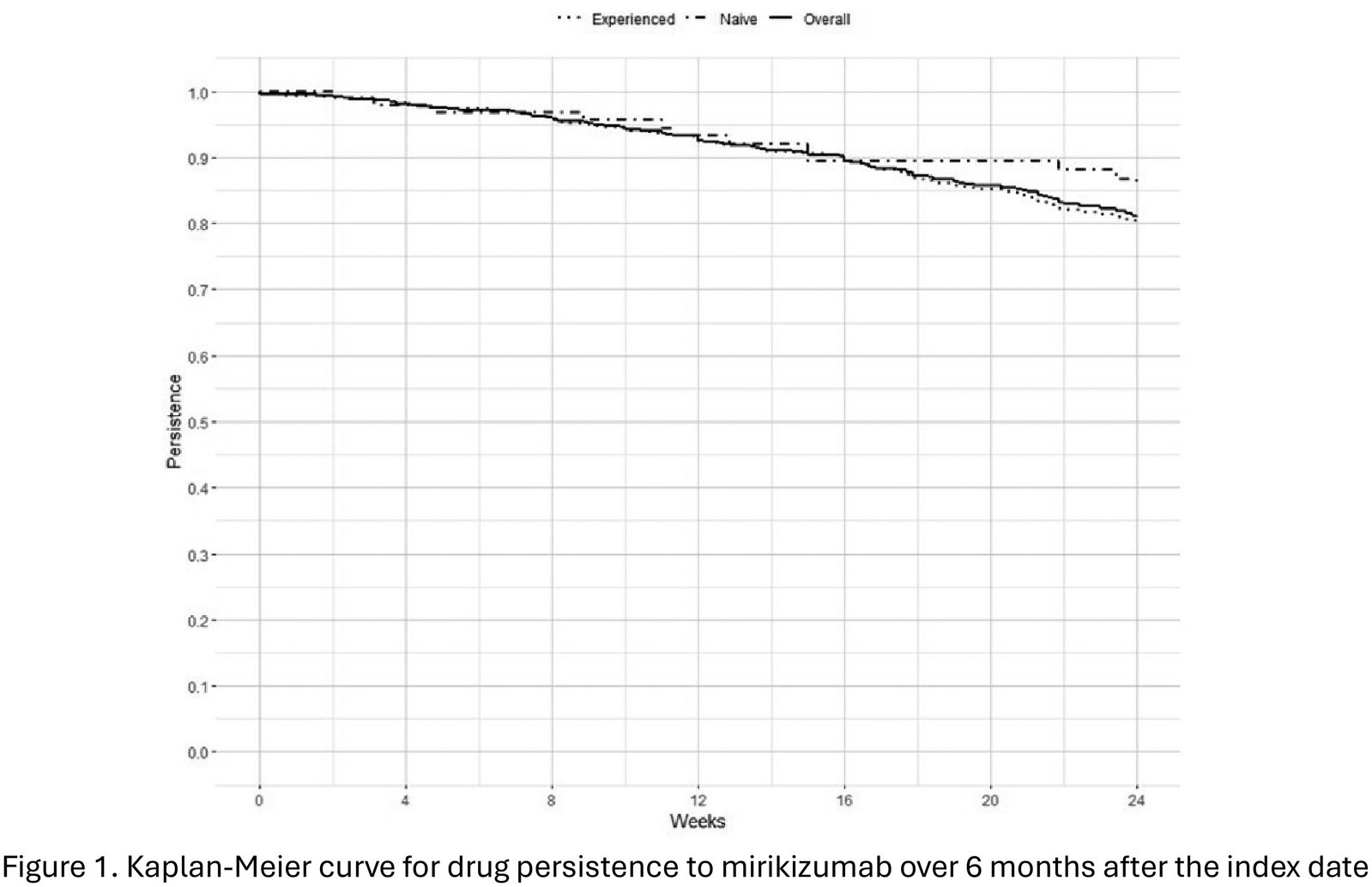

**Funding Agencies:**

Eli Lilly Canada Inc.

